# *Peltigera frigida* Lichens and Their Substrates Reduce the Influence of Forest Cover Change on Phosphate Solubilizing Bacteria

**DOI:** 10.3389/fmicb.2022.843490

**Published:** 2022-06-28

**Authors:** Cecilia Muster, Diego Leiva, Camila Morales, Martin Grafe, Michael Schloter, Margarita Carú, Julieta Orlando

**Affiliations:** ^1^Laboratory of Microbial Ecology, Facultad de Ciencias, Universidad de Chile, Santiago, Chile; ^2^Institute of Biology, University of Graz, Graz, Austria; ^3^Research Unit Comparative Microbiome Analysis, Helmholtz Zentrum München, Neuherberg, Germany; ^4^Millennium Institute Biodiversity of Antarctic and Subantarctic Ecosystems (BASE), Santiago, Chile

**Keywords:** Chilean Patagonia, lichen microbiome, *Nothofagus* forests, phosphorus cycling, *Peltigera*

## Abstract

Phosphorus (P) is one of the most critical macronutrients in forest ecosystems. More than 70 years ago, some Chilean Patagonian temperate forests suffered wildfires and the subsequent afforestation with foreign tree species such as pines. Since soil P turnover is interlinked with the tree cover, this could influence soil P content and bioavailability. Next to soil microorganisms, which are key players in P transformation processes, a vital component of Patagonian temperate forest are lichens, which represent microbial hotspots for bacterial diversity. In the present study, we explored the impact of forest cover on the abundance of phosphate solubilizing bacteria (PSB) from three microenvironments of the forest floor: *Peltigera frigida* lichen thallus, their underlying substrates, and the forest soil without lichen cover. We expected that the abundance of PSB in the forest soil would be strongly affected by the tree cover composition since the aboveground vegetation influences the edaphic properties; but, as *P. frigida* has a specific bacterial community, lichens would mitigate this impact. Our study includes five sites representing a gradient in tree cover types, from a mature forest dominated by the native species *Nothofagus pumilio*, to native second-growth forests with a gradual increase in the presence of *Pinus contorta* in the last sites. In each site, we measured edaphic parameters, P fractions, and the bacterial potential to solubilize phosphate by quantifying five specific marker genes by qPCR. The results show higher soluble P, labile mineral P, and organic matter in the soils of the sites with a higher abundance of *P. contorta*, while most of the molecular markers were less abundant in the soils of these sites. Contrarily, the abundance of the molecular markers in lichens and substrates was less affected by the tree cover type. Therefore, the bacterial potential to solubilize phosphate is more affected by the edaphic factors and tree cover type in soils than in substrates and thalli of *P. frigida* lichens. Altogether, these results indicate that the microenvironments of lichens and their substrates could act as an environmental buffer reducing the influence of forest cover composition on bacteria involved in P turnover.

## Introduction

Lichens are important components of the cryptogamic biota in forest ecosystems and represent interesting ecological niches for microorganisms. Beyond the classical definition of lichens, which includes a mutualistic symbiotic association between a fungus (mycobiont) and one or more photosynthetic microorganisms (photobiont/s; Nash, 2008), these organisms also harbor an associated microbiome (Cernava et al., 2015; Leiva et al., 2016, 2021; Aschenbrenner et al., 2017; Hawksworth and Grube, 2020). In recent years, lichen-associated bacteria have been described as forming a highly structured biofilm covering the lichen thallus (Grube and Berg, 2009; Bates et al., 2011; Hodkinson et al., 2012), which can play an essential role in lichens by facilitating, for example, the supply of crucial nutrients and therefore making their survival and growth possible in nutrient-poor substrates (Grimm et al., 2021). Besides, these bacterial communities associated with lichens could play an essential role in forest nutrition because of their potential to express enzymes driving carbon turnover, including cellulase, xylanase, and amylase, as well as their ability to fix nitrogen and solubilize phosphate (González et al., 2005; Liba et al., 2006; Selbmann et al., 2010; Cardinale et al., 2012; Sigurbjörnsdóttir et al., 2016; Almendras et al., 2018a,b). These bacterial communities associated with the lichen thallus are more than only an extension of those found in the substrate where the lichens grow, but an additional component of the traditional symbiosis, mainly due to the dissimilarity in abundance and the presence of specific bacterial groups throughout different lichens (Bates et al., 2011; Cardinale et al., 2012; Printzen et al., 2012; Leiva et al., 2016, 2021).

Phosphorus (P) is one of the most critical macronutrients in all ecosystems, and consequently, P availability in soils also affects the growth and performance of lichens. Microorganisms, including bacteria, play an important role by making P bioavailable (Richardson et al., 2001; Sashidhar and Podile, 2010; Chhabra et al., 2013; Sharma et al., 2013; Zhao et al., 2014). The way used by microorganisms to increase P bioavailability depends on phosphate bindings with organic and mineral molecules. The solubilization of organic phosphate involves several key enzymes, including alkaline and acid phosphatases, phytases, and phosphonatases (Rodríguez et al., 2006; Sharma et al., 2013). Mineral phosphate solubilization depends on the production of organic acids, which is essential for the dissolution of many poorly soluble mineral phosphates. The most studied organic acid is gluconic acid, produced by the membrane-bound glucose dehydrogenase, which catalyzes the direct oxidation of glucose (Goldstein, 1995). In forests, litterfall is the primary route of nutrient returns to the soil, releasing nutrients by decomposition (Osman, 2013). However, different plant species differ in the amount and quality of organic matter they add to the soil (Hooper et al., 2000; Finér et al., 2007; Šnajdr et al., 2013). Therefore, tree species may differently influence soil chemical properties, such as the pH or the relative content and chemical form of macronutrients (Binkley and Valentine, 1991; Augusto et al., 2000; Frouz et al., 2009; Iovieno et al., 2010). For example, P fluxes in coniferous and deciduous forests differ, with the concentration of P in the forest floor under coniferous trees being about three times higher than in deciduous forests, probably because the P content of coniferous foliage is larger than in deciduous one (Sohrt et al., 2017).

*Peltigera frigida* is one of the most conspicuous lichens in forests of the Coyhaique National Reserve in Aysén Region (Leiva et al., 2016), but its abundance is low in other parts of Southern Chile (Ramírez-Fernández et al., 2013; Zúñiga et al., 2015). It has been proposed that its adaptation could be partly due to the peculiarities of its microbiome (Leiva et al., 2021), as it could be related to soil properties due to vegetation composition and historic events suffered by the study site (Fajardo and Gundale, 2015). In particular, the main events affecting the study site were wildfires and subsequent reforestation with pines, which are reported as crucial drivers of phosphorus availability (Dickie et al., 2014; Butler et al., 2018).

Based on this information, we asked if the microenvironments associated with the terricolous cyanolichen *P. frigida* (thallus and substrate) reduce the influence of the tree cover type on the diversity of phosphate solubilizing bacteria (PSB). This question is driven by previous studies showing that *P. frigida* thalli and substrates constitute different microenvironments that select specific bacterial communities (Leiva et al., 2016, 2021), which could reduce the impact that the tree cover type exerts on the diversity of PBS in soils without lichen influence. In order to answer this question, we investigated the diversity of PSB along a gradient including sampling sites from mature native forest to second-growth forests afforested with exotic pines in the Coyhaique National Reserve (Aysén Region, Chile), considering three microenvironments related to the forest floor: (i) *P. frigida* thalli, (ii) their associated substrate (i.e., the soil beneath each thallus), and (iii) forest soil without lichen influence.

## Materials and Methods

### Selection and Characterization of Sampling Sites

The Coyhaique National Reserve (45°31′42.96”S, 72°1′51.95”W) is located in the Aysén Region of Southern Chile. In this reserve, deciduous forests with a predominance of *Nothofagus pumilio* constitute the typical vegetation. The native mature forests were affected by several wildfires in the region between 1930 and 1950 (Quintanilla Pérez, 2007; Fajardo and Gundale, 2015), after which natural regeneration resulted in native second-growth forests. Also, to protect the soil from erosion, plantations of various kinds of pines (mainly *Pinus contorta*) were part of afforestation programs organized by local authorities. However, the study area is not currently exposed to regular management practices since it is a protected area, and then the current forest community is the result of long-standing colonization.

Five ~200 m transects away ~300 m from each other (S1 to S5) were selected along a gradient in the forest cover composition present in the reserve trails (Supplementary Figure S1).

Forest cover at each site was estimated using the middle of the intercept point (Goodall, 1952) and the point-centered quarter (Mitchell, 2010) methods. The first one was used to calculate the relative coverage (*RC*) of each species in 2 m height, according to the formula $RC_{i}=\frac{n_{i}}{N_{T}}$, where *n*_i_ is the frequency of the species *i* and *N_T_* is the number of sampling points per site (Supplementary Table S1). The second method was used to calculate various indices from the field-collected data for each site (Supplementary Tables S2–S6). In this method, a reference point was selected every 20 m and divided into four quadrants. The closest tree was described at each of these quadrants by three parameters: species, distance to the reference point (*d_i_*), and circumference at breast height (*CBH*). Then, three basic values were calculated: the radius of the tree trunk (*r*), according to the formula $r_{i}=\frac{CBH_{i}}{2\cdot \pi }$; the basal area covered by each tree with the formula $A_{i}=\pi \cdot r_{i}{}^{2}$; and the total density (*TD*) of each species following TDi=1(diQ)2, where *Q* is the total number of sampled trees. After this, the relative density (*RD*) of each species was calculated with the formula $RD_{i}=\frac{n_{i}}{Q}$, where *n_i_* corresponds to the total number of trees of each species. The absolute density (*D*) was calculated dividing the relative density by the total density according to $D_{i}=\frac{RD_{i}}{TD_{i}}$. Additionally, five other indices were calculated by the point-centered quarter method (Supplementary Table S7). The frequency (*F*) was calculated according to the formula $F_{i}=\frac{j_{i}}{k}$, where *j_i_* is the total number of points of each species, and *k* is the number of sampled points. Then, the relative frequency (*RF*) was calculated by dividing the *F* value of each species by the sum for all three species, according to $RF_{i}=\frac{F_{i}}{\sum F}$. The coverage (*C*) of each species was calculated with the formula $C_{i}=\frac{A_{i}\cdot D_{i}}{n_{i}}$, and the relative coverage (*RC*) by $RC_{i}=\frac{C_{i}}{\sum C}$. Finally, the importance value (*IV*) was calculated according to the formula $IV_{i}=RF_{i}+RC_{i}+RD_{i}$, where the relative density (*RD*) of each species was calculated with the formula $RD_{i}=\frac{n_{i}}{Q}$, where n_i_ corresponds to the total number of trees of each species and *Q* is the total number of sampled trees. The importance value was relativized as Relative Importance (proportion of the total importance value) as it is easier to compare.

### Samples Collection

From the transect defined at each study site, five *Peltigera frigida* thalli and their associated substrates (Supplementary Table S8), alongside five soil samples from the surroundings (Supplementary Table S9), were collected. The lichen-associated samples were collected at least 1 m from the next closest to minimize the resampling of the same genetic individual. Besides, the soil samples were collected at least 1 m away from any lichen. The samples were placed in paper bags and transported in cooled containers. In the laboratory, the thalli were separated from the attached substrate with sterile brush and spatula. Thallus samples were stored in paper bags at room temperature, while the substrate and soil samples were sieved and stored in plastic tubes at −20°C.

### Physicochemical Parameters

From each soil sample, physicochemical parameters were determined as follows: pH was measured by potentiometry using a 1:2 ratio in 1 M KCl. Moisture content (MC; Steubing et al., 2002) and total organic matter (TOM; Sadzawka et al., 2006) were calculated gravimetrically before and after desiccation and calcination, respectively. Nitrate (N-NO_3_^−^), ammonium (N-NH_4_^+^), and phosphorus (P Bray) concentrations were measured spectrophotometrically from 1:10 soil extracts in deionized water, 1 M KCl, and Bray 1 extract, respectively (Bray and Kurtz, 1945; Steubing et al., 2002). Phosphorus sequential fractionation was determined by following Hedley’s protocol (Hedley et al., 1982) with the modification of do Nascimento et al. (2015). The soil was sequentially extracted with distilled water, 0.5 M NaHCO_3_ (pH 8), 0.1 M NaOH, and mineral phosphorus (Pm) was calorimetrically quantified (Murphy and Riley, 1962). Subsequently, all samples were digested with H_2_SO_4_ and K_2_S_2_O_8_ to determine organic phosphorus (Po) as the difference between total and mineral phosphorus (Po = Pt-Pm).

### DNA Extraction

DNA was extracted from thallus, substrate, and soil samples using the DNeasy PowerSoil Kit (Qiagen), with 0.25 g per soil and substrate samples, as suggested by the manufacturer, and 0.15 g per thallus sample according to pre-experiments performed (data not shown). Modifications compared to the standard protocol included an overnight incubation at 4°C after adding the precipitation solution C2 (step 8) and an increase in time from 30 s to 1 min for the centrifugation steps after bead beating (step 6), ethanol washing (step 16), and elution (step 21).

### Identification of the Lichen Symbionts

From the DNA of lichen thalli, mycobionts were identified by analyzing the fungal 28S rDNA region amplified with primers LIC24R (Miadlikowska and Lutzoni, 2000) and LR7 (Vilgalys and Hester, 1990), and the ITS region amplified with primers ITS1F (Gardes and Bruns, 1993) and ITS4 (White et al., 1990). In addition, photobionts were identified using the cyanobacterial 16S rDNA region with the primers PCR1 and PCR18 (Wilmotte et al., 1993). All amplicons were sequenced with the forward primers using the Genetic Analyzer 3730XL (Applied Biosystems) using a sequencing service (Macrogen, Seoul, Korea).

Mycobiont identity was confirmed by a *de novo* phylogenetic analysis of the 28S and ITS sequences, carried out on the T-BAS web platform using the *Peltigera* reference tree (Carbone et al., 2019). In the case of the cyanobionts, the evolutionary history was inferred using the Neighbor-Joining method. For this, evolutionary distances were calculated using Kimura’s 2-parameter method, as suggested by MEGA7 (Kumar et al., 2016). The analysis involved 55 nucleotide sequences, including those from this work and reference sequences obtained from both cyanolichens and free-living cyanobacteria. All positions containing deletions were removed. Evolutionary analyses were performed in the software MEGA7 (Kumar et al., 2016). Finally, the phylogenetic tree was edited in iTOL (Letunic and Bork, 2019).

### Quantification of Genes Related to Phosphate Solubilization

Using the DNA obtained from the thallus, substrate, and soil samples, quantification of the bacterial markers for phosphate solubilization, including the quinoprotein glucose dehydrogenase gene (*gcd*), alkaline phosphatase gene (*phoD*), acid phosphatase gene (*phoN*), phytase gene (*appA*), and phosphonoacetaldehyde hydrolase gene (*phnX*), was performed using quantitative PCR (qPCR) on an ABI 7300 Real-time PCR System (Kurth et al., 2020) and specific primers for each gene (Bergkemper et al., 2016a). These primers were reported as specific for bacteria, except *phoD*, which amplified genes related to some fungi but not *Peltigera* (Bergkemper et al., 2016a). Therefore, hereinafter we refer to phosphate solubilizing bacteria (PSB), although we cannot discard the presence of fungal genes related to P turnover. In addition, the 16S rRNA gene was also quantified as a marker for bacterial abundance (Kurth et al., 2020).

After each qPCR run, a melting curve analysis was performed to verify the amplicon specificity. The quantification of the target gene was conducted by using serial dilutions of plasmid-encoding the target genes (10^7^ to 10^1^ gene copies μl^−1^). Details on the used standards were published previously (Bergkemper et al., 2016b). All samples were diluted 1:4 to avoid inhibition during amplification caused by co-extracted humic substances based on a previous dilution test (data not shown). The efficiency of the qPCR was calculated as Efficiency (%) = [10(−1/slope) − 1]. Values were always above 85%, while the R^2^ of the standard curve was above 0.99.

### Data Analysis

The abundance data of phosphate solubilization genes were log10 transformed and subjected to the Shapiro–Wilk test. The abundance of genes and physicochemical parameters were compared using one-way ANOVA and Tukey’s multiple comparison test. Non-metric multidimensional scaling (NMDS) analysis and pairwise Adonis test were performed based on Bray-Curtis distance, while redundancy analysis (RDA) was based on Euclidean distance and a stepwise selection of environmental variables using the ordiR2step function. All statistical tests and graphics were performed in R Studio (R version 3.5.3) using the vegan (Oksanen et al., 2017) and ggplot (Wickham, 2016) packages.

## Results

### Characterization of the Study Sites

For this study, we selected five sampling sites representing different tree cover types. The first transect is located in a native *N. pumilio* mature forest (S1); the second one is spanning the transition between the native mature forest and a native second-growth forest (S2); the third one is located in the second-growth forest (S3); the fourth one is in the transition between the native second-growth forest and a second-growth forest of *N. pumilio* with the presence of pine specimens (S4); and finally, the last transect is the native second-growth forest with the presence of pine specimens (S5).

The first method used to estimate the forest cover, the middle of the intercept point method, calculates the relative coverage of the canopy of a tree species at a certain height (in this case, 2 m). The sum of this variable can reach values greater than 1 since the canopies of the trees overlap. According to this method, *N. pumilio* had the highest relative tree canopy coverage in all five sites. The sites S2 and S3, with second-growth forests, showed a less relative coverage of the tree canopy of *N. dombeyi*. Finally, the presence of *P. contorta* displaced the relative coverage of *N. dombeyi* in sites S4 and S5 (Table 1). The second method, the point-centered quarter method, calculates relative importance as the sum of the relative density, relative frequency, and relative volume for each species, being a way of measuring tree dominance. According to this method, *N. pumilio* is the most dominant tree species in all the forest sites, but it decreases in S4 with a concomitant increase of *N. dombeyi*, while *P. contorta* increased its dominance from S4 to S5 (Table 1). In summary, from S1 to S5, there is a decrease in the coverage of *N. pumilio* and an increase in that of *P. contorta*. *N. dombeyi* was also present but with no apparent coverage gradient among the sites (Table 1).

**Table 1:** Forest cover of the arboreal species present in the different sites (*Nothofagus pumilio*, *Nothofagus dombeyi,* and *Pinus contorta*).

		S1	S2	S3	S4	S5
Relative Coverage	*N. pumilio*	0.90	0.94	0.88	0.82	1.00
	*N. dombeyi*	0.75	0.63	0.69	0.65	0.18
	*P. contorta*	0.00	0.00	0.00	0.24	0.41
Relative Importance	*N. pumilio*	0.80	0.77	0.81	0.58	0.72
	*N. dombeyi*	0.20	0.23	0.19	0.30	0.09
	*P. contorta*	0.00	0.00	0.00	0.12	0.19

The mean values of two metrics per site are included: relative coverage and relative importance of tree canopies.

The physicochemical characteristics of the soils of the five sites are summarized in Table 2. Total organic matter, P Bray, soluble Pm (Pm-H_2_O), labile Pm (Pm-HCO_3_^−^), and labile Po (Po-HCO_3_^−^) were significantly higher in S5 than in S1. Notably, soluble Pm was about 15-fold higher, while P-Bray was 3-fold higher in S1 than S5. Conversely, moisture content and N-NO_3_^−^ tend to significantly decrease from S1 to S5. Finally, pH was higher in S2 and S4 than in S3 and S5, while N-NH_4_ and Po-NaOH were similar in all sites.

**Table 2:** Physicochemical parameters of the soil samples from the different sites.

Moisture Content (%)	S1				S2				S3				S4				S5			
	57.7	±	31.2	b	44.3	±	25.3	ab	42.7	±	1.3	ab	16.7	±	0.9	a	23.0	±	0.3	a
Total organic matter (%)	25.9	±	4.6	a	21.6	±	2.5	a	44.6	±	10.5	ab	28.9	±	10.2	ab	52.5	±	23.9	b
pH (H_2_O)	5.4	±	0.05	ab	5.5	±	0.1	b	5.4	±	0.1	a	5.6	±	0.1	b	5.3	±	0.1	a
N-NO_3_^−^ (mg/kg)	40.8	±	18.8	b	1.8	±	1.1	a	77.1	±	10.4	c	10.6	±	6.7	a	41.5	±	5.7	b
N-NH_4_^+^ (mg/kg)	5.7	±	5.2	a	6.3	±	5.4	a	3.8	±	2.0	a	4.1	±	0.4	a	2.2	±	1.9	a
P Bray (mg/kg)	21.2	±	16.0	ab	5.8	±	2.1	a	49.8	±	28.9	bc	17.4	±	8.8	ab	71.4	±	26.5	c
Pm-H_2_0 (mg/kg)	2.5	±	0.7	a	3.6	±	1.0	a	0.8	±	0.2	a	3.9	±	0.9	a	36.8	±	12.6	b
Pm-HCO_3_^−^ (mg/kg)	67.7	±	34.8	ab	33.5	±	18.6	b	72.4	±	13.6	ab	84.8	±	20.6	a	172.4	±	20.4	c
Po-HCO_3_^−^ (mg/kg)	65.2	±	35.3	ab	58.2	±	36.1	ab	94.4	±	1.9	b	37.5	±	10.4	a	150.8	±	6.7	c
Pm-NaOH (mg/kg)	358.5	±	152.0	ab	156.5	±	19.5	ab	110.2	±	11.2	a	256.2	±	33.2	ab	420.5	±	308.5	b
Po-NaOH (mg/kg)	165.5	±	62.8	a	208.0	±	12.8	a	162.6	±	17.5	a	394.9	±	197.6	b	195.7	±	80.7	a

Each value is the mean ± SD of five replicates. Different letters represent a significant difference (*p* < 0.05; one-way ANOVA) between sites. Soluble Pm (Pm-H_2_0), labile Pm (Pm-HCO_3_), labile Po (Po-HCO_3_), moderately labile Pm (Pm-NaOH), and moderately labile Po (Po-NaOH).

### Abundance of Phosphate Solubilization Marker Genes

Five specimens of *P. frigida* thalli were selected from each of the study sites. The identification of the mycobionts was confirmed by a phylogenetic analysis based on the 28S rRNA gene (Supplementary Figure S2) and the ITS hypervariable region (ITS-HR; Miadlikowska et al., 2003; Supplementary Figure S3). Further, all specimens selected were associated with the same photobiont haplotype namely *Nostoc* sp. C01 (Zúñiga et al., 2015) according to a 16S rRNA gene-based phylogenetic analysis (Supplementary Figure S4).

**Figure 1:**
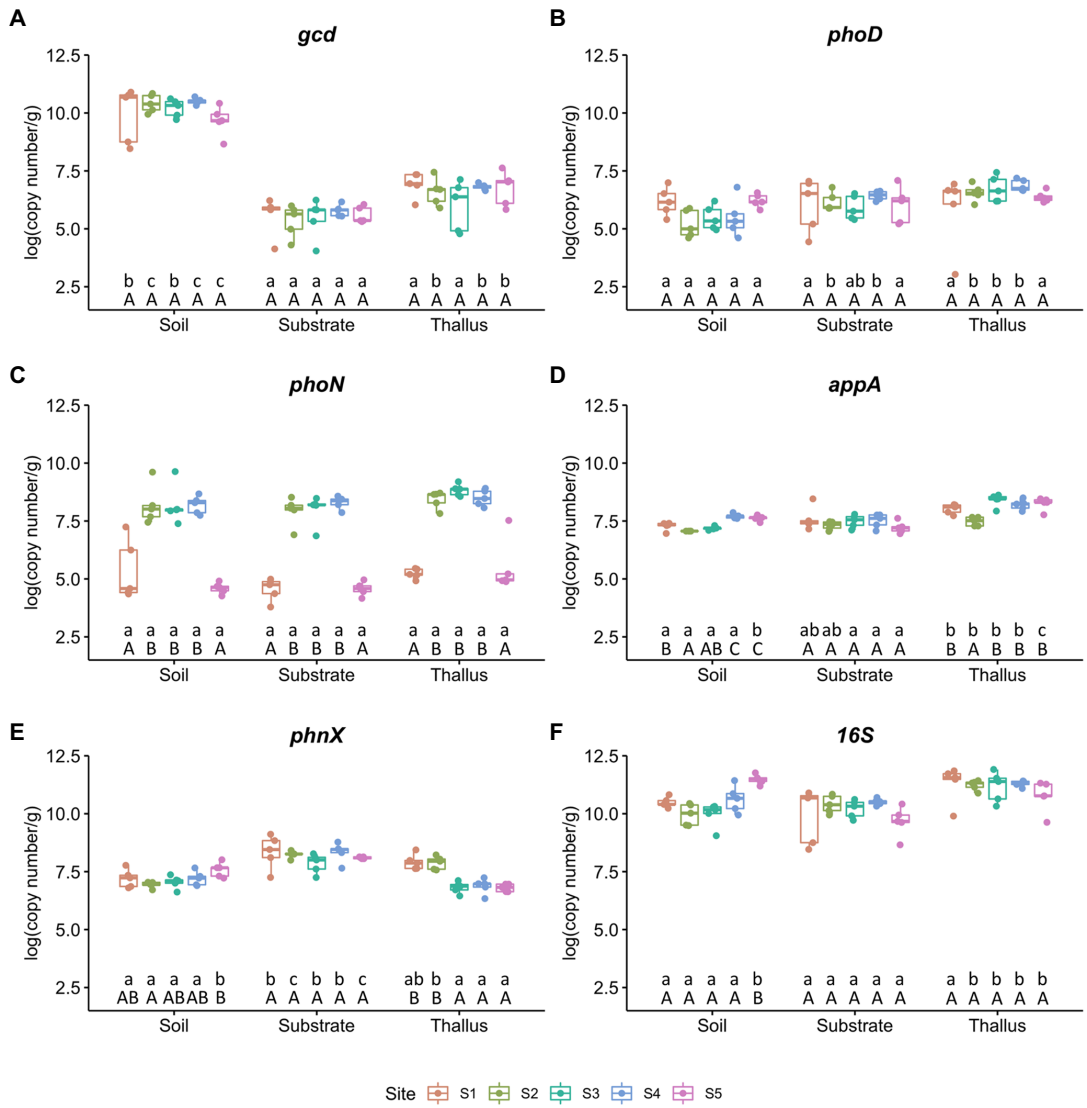
Abundance of phosphate solubilization markers per site. Each value is the mean ± SD of five replicates. Different lowercase and capital letters represent significant difference (*p* < 0.05; one-way ANOVA) between microenvironments and sites, respectively. **(A)** Quinoprotein glucose dehydrogenase gene (*gcd*). **(B)** Alkaline phosphatase gene (*phoD*). **(C)** Acid phosphatase gene (*phoN*). **(D)** Phosphonatase gene (*appA*). **(E)** Phosphonoacetaldehyde hydrolase gene (*phnX*). **(F)** 16S rRNA gene as marker for bacterial abundance.

From the samples of the three different microenvironments (i.e., *P. frigida* thalli, substrates, and the surrounding soils) collected from the five sites (i.e., S1 to S5), we measured by qPCR the relative abundance of one gene that codes for an enzyme that triggers mineral phosphate solubilization (*gcd*), four genes that codes for enzymes involved in organic phosphate solubilization (*phoD*, *phoN*, *appA*, *phnX*), and the 16S rRNA gene as a bacterial marker (Figure 1). The quinoprotein glucose dehydrogenase gene (*gcd;* Figure 1A) was the most abundant marker in all soil samples, and unexpectedly its abundance was ~40,000 times higher in soils than in substrates and ~ 3,000 times higher in soils than in thalli in all the study sites. However, there were no significant differences in the abundance of this marker gene comparing the same microenvironment among the sampling sites. The abundance of the alkaline phosphatase marker gene (*phoD;* Figure 1B) also did not show differences when comparing the sampling sites, and although in the microenvironments of the extreme sites (i.e., S1 and S5) its abundance did not show significant differences, in the soil samples of the intermediate sites its abundance was significantly lower than in substrates and thalli. On the contrary, the abundance of the acid phosphatase marker gene (*phoN;* Figure 1C) was significantly less abundant in the extreme sites (i.e., S1 and S5) than in the other sites, independent of the microenvironment. The abundance of the phytase marker gene (*appA;* Figure 1D) and the phosphonoacetaldehyde hydrolase marker gene (*phnX;* Figure 1E) had the highest variation in soils throughout the sites, but *appA* showed the highest abundance in thalli and *phnX* in substrates. Finally, the 16S rRNA marker gene (Figure 1F) abundance almost did not change among sites, except for the soil samples from S5 that presented the highest abundance for this microenvironment. However, the abundance of the bacterial marker gene was significantly higher in thalli than in soils and substrates from the intermediate sites (i.e., S2 to S4).

### Relation of Explanatory Variables With the Diversity of Phosphate Solubilizing Marker Genes

**Figure 2:**
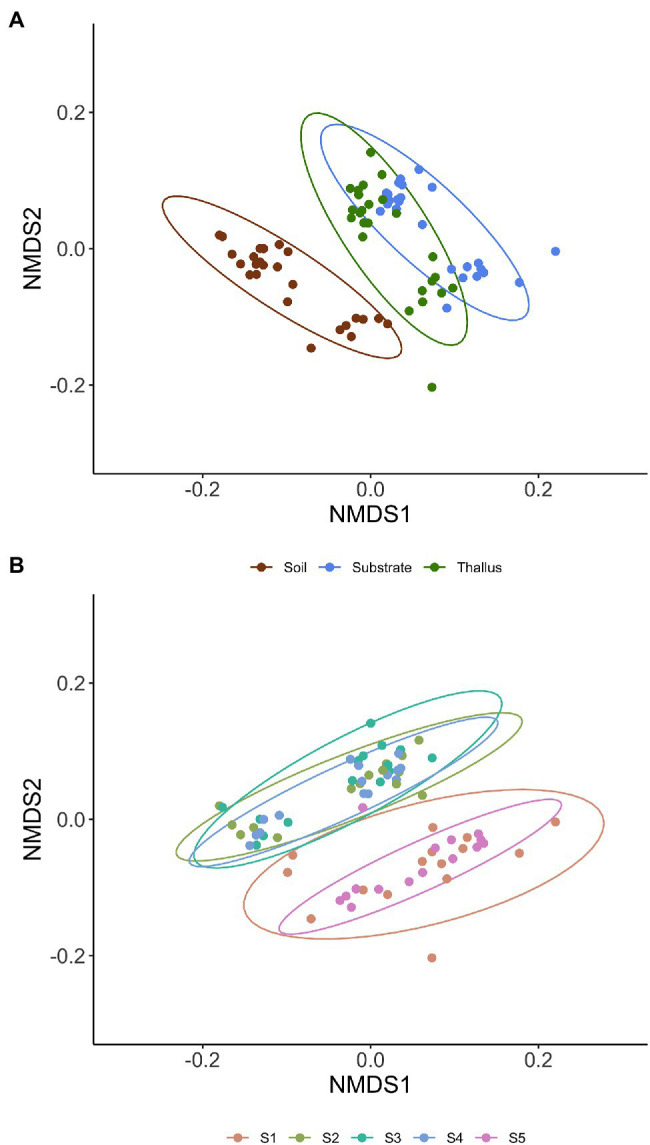
Non-metric multidimensional scaling (NMDS) multivariate analysis of the diversity of phosphate solubilization marker genes based on Bray-Curtis distance. Ellipses of 95% confidence are included for enclosing samples from each microenvironment (**A**; i.e., thallus, substrate, and soil) and sites (**B**; i.e., S1 to S5).

NMDS multivariate analysis of the diversity of phosphate solubilization marker genes shows a clear separation of soil samples from those of lichen-associated microenvironments (i.e., substrates and thalli) in all the sampling sites (Figure 2A; Table 3). On the contrary, the separation of substrate and thallus samples, although statistically significative, is partially overlapped, being more evident in the intermediate than in the extreme sampling sites (i.e., S1 and S5). When comparing the diversity in the different sampling sites (Figure 2B; Table 4), there are two well-defined groups, the first one including S2, S3 and S4, and the second one including S1 and S5. Notoriously, the 95% ellipse encompassing the points of S1 is the largest one and encloses the 95% ellipse of S5, which is noticeably smaller.

**Table 3:** PERMANOVA (*adonis* function in R) to evaluate the effect of the microenvironment on the distribution of the variance of each sample type.

	S1		S2		S3		S4		S5	
	R^2^	*value of p*	R^2^	*value of p*	R^2^	*value of p*	R^2^	*value of p*	R^2^	*value of p*
Soil vs. Substrate	0.52052	**0.009**	0.87044	**0.009**	0.78180	**0.006**	0.85770	**0.005**	0.82316	**0.009**
Soil vs. Thallus	0.54876	**0.007**	0.88229	**0.011**	0.84584	**0.006**	0.84278	**0.007**	0.50864	**0.009**
Substrate vs. Thallus	0.28023	**0.067**	0.42550	**0.012**	0.38214	**0.016**	0.80624	**0.006**	0.35070	**0.028**

Significant *value of p* is shown in bold.

**Table 4:** PERMANOVA (*adonis* function in R) to evaluate the effect of the site on the distribution of the variance of each sample type.

	Soil		Substrate		Thallus	
	R^2^	*value of p*	R^2^	*value of p*	R^2^	*value of p*
S1 vs. S2	0.51529	**0.005**	0.46665	**0.013**	0.58596	**0.009**
S1 vs. S3	0.46250	**0.006**	0.43940	**0.010**	0.65613	**0.007**
S1 vs. S4	0.51017	**0.009**	0.50992	**0.009**	0.65073	**0.013**
S1 vs. S5	0.28053	**0.038**	0.04938	0.772	0.28545	**0.026**
S2 vs. S3	0.04989	0.782	0.04133	0.889	0.40400	**0.007**
S2 vs. S4	0.30240	**0.022**	0.14294	0.317	0.47407	**0.011**
S2 vs. S5	0.84686	**0.013**	0.66404	**0.010**	0.65439	**0.006**
S3 vs. S4	0.23131	0.054	0.17263	0.170	0.17899	0.304
S3 vs. S5	0.81610	**0.009**	0.60228	**0.008**	0.53371	**0.009**
S4 vs. S5	0.79479	**0.008**	0.77495	**0.009**	0.57656	**0.005**

Significant *value of p* is shown in bold.

To determine the effect of the edaphic and tree cover parameters on the abundance of phosphate solubilizing genes, we performed redundancy analyses (RDA) and ordistep for selecting the variables that significantly contribute to the model. The RDA analysis of soil samples, which explained 60.1% of the total variance, corroborates that sites S2, S3, and S4 grouped closely together and that S1 is the most diverse of all sites, in contrast to S5, which is the less diverse (Supplementary Figure S5A). The variables that significantly contributed to this model were the relative abundance of tree canopies of *N. pumilio*, *N dombeyi*, and *P. contorta*, pH, and the labile fraction of inorganic mineral phosphorus (Pm-HCO_3_^−^; Table 5). On the other hand, the variance explained by the RDA analyses for substrates (Supplementary Figure S5B) and thalli (Supplementary Figure S5C) are not robust enough (36.7 and 47.5% of the total variance explained, respectively) to appropriately assert patterns of phosphate solubilizing bacteria diversity. In these cases, the only significant explanatory variables were the relative abundance of *N. pumilio* tree canopies and the labile fraction of mineral phosphorus (Pm-HCO_3_^−^), respectively (Table 5).

**Table 5:** Stepwise selection of significant environmental factors explaining the abundance variation of phosphate solubilization and mineralization markers.

	Df	AIC	F	*value of p*
**Soil**				
C *P. contorta*	1	38.634	10.2	0.005
C *N. pumilio*	1	37.064	3.4	0.015
C *N. dombeyi*	1	36.082	2.7	0.030
pH	1	34.860	2.8	0.030
Pm-HCO_3_^−^	1	33.371	2.8	0.030
**Substrate**				
C *N. pumilio*	1	44.947	2.8	0.040
**Thallus**				
Pm-HCO_3_^−^	1	44.480	3.3	0.010

Results are based on RDA ordination using ordistep function in R.

## Discussion

We assessed the abundance of phosphate solubilizing bacteria (PSB) in three microenvironments related to the forest floor: (i) *Peltigera frigida* thalli, (ii) their associated substrate (i.e., the thallus subjacent soil), and (iii) the forest soil. These habitats were assessed in five sites with different tree cover in the Coyhaique National Reserve of the Chilean Patagonia characterized by temperate forests of *Nothofagus* species (Donoso, 1993; Veblen et al., 1996; Fajardo and González, 2009). We selected five sites representing: a native mature forest (S1), a native second-growth forest (S3), a native second-growth forest with the presence of pine specimens (S5), and the transition zones between these vegetative formations (S2 and S4, respectively). According to the methods used to calculate the forest coverage, *N. pumilio* was the dominating tree species with the greatest relative importance and coverage at all sites, and *P. contorta* was increasing its abundance in the last two sites (S4 and S5) with a decrease of *N. dombeyi*.

### The Abundance of PSB in Forest Soils Is Affected by the Tree Cover Type

Changes in the soil P content are influenced by multiple and diverse factors. One of them are wildfires, which in Patagonian forests has been reported to have long-term effects on soil properties, such as decreases in organic C and N and increases in pH and extractable P (Alauzis et al., 2004). Another factor is the influence of trees on soil chemical properties through their root exudates and the loss of their leaves in deciduous species (such as *N. pumilio*), contributing to changes in pH and the relative content and availability of macronutrients (Binkley and Valentine, 1991; Augusto et al., 2000; Frouz et al., 2009; Iovieno et al., 2010). Additionally, enzymatic activities of phosphorus solubilizing microorganisms could also affect the soil P content, which not only responds to the soil P availability but also to the balance with other nutrients, mainly nitrogen (Sorkau et al., 2018). In fact, Margalef et al. (2017) showed that the availability of nitrogen in soil is the main factor explaining phosphatase activity in temperate climates.

The quantification of the PSB marker genes in the soil samples evidenced two noticeable results. The first one was the high abundance of *gcd*, which codes an enzyme that allows the direct oxidation of glucose to gluconic acid, one of the main mechanisms of extracellular Pm solubilization studied in bacteria (Sharma et al., 2013; Sindhu et al., 2014; An and Moe, 2016). Therefore, the solubilization through gluconic acid of Pm from mineral complexes found in soils would be important in the sampling sites. There are other organic acids such as malic, lactic, citric and oxalic acids, which can also solubilize Pm (Sharma et al., 2013), but they have been less studied in this regard because the enzymes associated with their production play different intracellular roles and could be not relevant in the P cycle (Grafe et al., 2018). The second one was the pattern of abundance of the acid phosphatase marker gene (*phoN*), which was lower in the extreme sampling sites (i.e., S1 and S5) and more similar to each other than to the abundance in the intermediate sites. This could be related, in part, to the higher values of available nitrogen (ammonium plus nitrate) measured in the extreme sampling sites regarding the transition sampling sites (i.e., S2 and S4). In addition, the intermediate sites (S2-S3-S4) correspond to sectors of young trees and, therefore, they have a more active growth than those in the mature forest (S1) and then a greater demand for phosphorus that could explain the higher abundance of this marker gene. The measured pH in the soil samples (5.3 to 5.6), suggests that the activity of the enzyme coded by *phoN* could be more relevant than the activity of the alkaline phosphatase (encoded by *phoD*), as has been shown for other forest soils (Staddon et al., 1998; Herbien and Neal, 2008).

On the other hand, the growth dynamics of the tree species present in the sampling sites is different. *P. contorta* is a fast-growing exotic conifer, which would demand higher amounts of nutrients, such as P, compared to the native species *N. pumilio* (Fajardo and Gundale, 2015). Our results are in accordance with a comparative study about the P flux in coniferous and deciduous forests of the northern hemisphere, where the concentration of P in the forest floor under coniferous trees was about three times higher than in deciduous forests (Sohrt et al., 2017). Since the P content of coniferous foliage is larger than in deciduous one (Sohrt et al., 2017) and litterfall plays a critical role in nutrient cycling (Attiwill and Adams, 1993), the tree cover could affect the abundance of phosphate solubilizing microorganisms in soils. Indeed, in our study, the tree cover type strongly affected the abundance of PSB in the forest soil, being the relative coverage of all three tree canopies identified as a significant driver. The most noteworthy result regarding the effect of the tree cover influence on the PSB marker genes is the reduced diversity detected in the site with the highest presence of pines (S5) compared with the site of native mature forest (S1), although in the former the number of bacteria is higher.

Other significant explanatory variables were the pH, reported as one of the main factors controlling bacterial communities in soils (Fierer and Jackson, 2006), and labile Pm, which could be the primary source of P for PSB (Sims and Pierzynski, 2005). pH was higher in the transition zones (S2 and S4) than in the native second-growth forests independently of the presence of pines (S3 and S5), but we found a significant increasing gradient of total organic matter and various phosphorus fractions from the native mature forest (S1) to the native second-growth forest with the presence of pine specimens (S5). Although the phosphorus content values in our samples were in the range of soils in southern Chilean forests (Redel et al., 2008), differences were still observed between the sites.

It is important to mention that other factors, such as rhizospheric exudates and the association with mycorrhizae, could affect soil P dynamics (Chen et al., 2008). Interestingly, all the tree species of our study associate with ectomycorrhizal fungi (Bradbury et al., 1998; Godoy and Marín, 2019), which are highly efficient in P turnover by combining a high capacity to explore soils, the formation of polyphosphates, and the activity of acid phosphatase and phytase enzymes (Chen et al., 2008). However, they are affected by forest anthropogenic disturbances such as fires and afforestation (Marín et al., 2017), which could explain our observations that some bacterial markers related to the solubilization of organic phosphate (e.g., *phoN*) were lower in the soils of the sites where ectomycorrhizae would be contributing more importantly to the P turnover (i.e., S1 and S5).

Our results reinforce the concept that microorganisms are susceptible to changes in environmental conditions and management practices; thus, they are good indicators for assessing changes in soil quality or soil recovery from disturbances (Quintanilla Pérez, 2007), such as the afforestation with exotic trees after wildfires. A deep understanding of microbial communities in forest habitats with different forest cover is then essential for predicting the response of forest ecosystems to changes in environmental conditions (Lladó et al., 2017).

### Lichen Microhabitats Shape Their Associated Bacteria, Reducing the Impact of the Tree Cover on the Abundance of PSB

Thalli and substrates could be considered microenvironments with a high level of nutrient turnover (N, P or C), as biological soil crusts (Kurth et al., 2021) or the rhizosphere (Kuzyakov and Blagodatskaya, 2015). According to our results, the solubilization of Po from stable molecules such as phytate (through the enzyme coded by *appA*) or phosphonate (through the enzyme coded by *phnX*) seems to be more important in these microenvironments, respectively, than the solubilization of Pm, which would be more relevant in soils due to the higher abundance of the *gcd* marker gene. Phytate is accumulated by plants (Balaban et al., 2016), and to a lesser extent by microorganisms (Turner et al., 2021), as a P storage compound. The capacity to hydrolyse phytate has been reported as widespread among lichens, which potentially contributes to P capture from atmospheric deposits and plant leachates (Higgins and Crittenden, 2015). Phosphonates, on the other hand, can be found in membranes or excretions of microorganisms (Horsman and Zechel, 2017; Calcott et al., 2018) and groups of phosphonate biosynthetic genes have been reported in lichenized *Nostoc* (Gagunashvili and Andrésson, 2018). Therefore, considering the close contact between the thallus and the substrate in foliose lichens, as *P. frigida*, that the secondary metabolites produced by the lichen would diffuse to its substrate (Leiva et al., 2016), and that the main photobiont of *Peltigera* lichens were detected in both thalli and substrates (Zúñiga et al., 2017), the lichen-related microenvironments could be a rich medium in this type of compounds.

As an essential element, P plays a relevant role as a limiting macronutrient in the growth and activity of lichens. For example, it has been observed that a single immersion in phosphate solution can double the annual growth of the lichen *Lobaria pulmonaria* (McCune and Caldwell, 2009), as well as that fertilization with P, could increase nitrogen fixation in the cyanolichens *Peltigera aphtosa* and *Peltigera polydactyla* (Weiss et al., 2005). Considering the relevance of P in the development of lichens, different studies have been focused on the possible role that the lichen microbiota would be playing in functions related to phosphate solubilization processes. Indeed, several publications report potential PSB in lichen thalli (Bates et al., 2012; Grube et al., 2015; Sigurbjörnsdóttir et al., 2015). Thus, despite the lower abundance of organic phosphate solubilizers in comparison to mineral phosphate solubilizers, the former could play an essential role in recycling the old parts of the thallus (Grube et al., 2015), releasing phosphate that members of the lichen symbiosis could use. It is important to highlight that phosphatase and phytase activity have been reported in several species of lichens (Whiteford et al., 2010; Higgins and Crittenden, 2015), attributed to lichen enzymes being produced by the mycobiont. However, the primers chosen for this study were designed to amplify bacterial genes (Bergkemper et al., 2016a), which would indicate that the lichen microbiome is at least contributing to P turnover. However, since the lichens of this study are terricolous (i.e., their substrate is soil), the high abundance of the *gcd* gene, as the molecular marker of mineral phosphate solubilizers, is in accordance with analyses of metagenomes of soils where this is the most abundant gene of a set of 40 genes that encode enzymes related to the P cycle (Bergkemper et al., 2016b; Grafe et al., 2018).

Previous studies have found that thalli and substrates constitute microenvironments that differentially structure the associated microbial communities (Grube and Berg, 2009; Bates et al., 2011; Hodkinson et al., 2012; Ramírez-Fernández et al., 2014; Leiva et al., 2016). In particular, a recent study has shown that the bacterial community associated with *P. frigida* thalli is more than a mere extension of the microbiota of their substrates, with several bacterial being highly abundant in lichens but almost absent in substrates (Leiva et al., 2021). In our study, some markers of P turnover were more abundant in thalli, which could be related to the fact that part of bacteria related to this function would be recruited from the substrates and would later be enriched in the thalli. This proposition is related to Cardinale et al. (2012), who suggests that those microbial groups with specific functions within the microbiome would be acquired from the new environment colonized by lichen propagules because they are better adapted to local conditions.

In contrast to soils, PSB in substrates and thalli were only correlated with *N. pumilio* cover and labile Pm, respectively, and RDA analysis did not explain much of the variance. This supports our proposal that the microenvironments of lichens and their substrates act as an environmental buffer reducing the influence of forest cover composition on bacteria involved in P turnover, and that other factors driving the association with lichens could be of importance, like microbiome specificity.

## Conclusion

Although the phosphorus turnover in forests is a complex process involving not only different organisms, such as plants, fungi, and bacteria, but also their interactions, altogether our results show that the diversity of genes related to phosphate solubilizing bacteria in forest soils is strongly affected by the tree cover. Conversely, lichens could act as an environmental buffer reducing the influence of forest cover composition on their associated bacteria involved in phosphorus turnover.

## Data Availability Statement

The original contributions presented in the study are included in the article/Supplementary Material; further inquiries can be directed to the corresponding author.

## Author Contributions

CeM, MC, and JO: conceptualization. MS, MC, and JO: funding acquisition. CeM, DL, CaM, and JO: methodology. CeM, DL, and MG: formal analysis. JO: project administration. MS and JO: supervision. All authors revised and approved the final version of the manuscript.

## Funding

This research was funded by ANID—National Fund for Scientific and Technological Development (FONDECYT) —1181510 and ANID—Millennium Science Initiative Program—ICN2021_002.

## Conflict of Interest

The authors declare that the research was conducted in the absence of any commercial or financial relationships that could be construed as a potential conflict of interest.

## Publisher’s Note

All claims expressed in this article are solely those of the authors and do not necessarily represent those of their affiliated organizations, or those of the publisher, the editors and the reviewers. Any product that may be evaluated in this article, or claim that may be made by its manufacturer, is not guaranteed or endorsed by the publisher.
