# Supplementary material for: Peltigera frigida Lichens and Their Substrates Reduce the Influence of Forest Cover Change on Phosphate Solubilizing Bacteria
Source: Front Microbiol. 2022 Jun 28;13:843490. doi: 10.3389/fmicb.2022.843490 (PMC9275751; doi:10.3389/fmicb.2022.843490)
Supplement: Supplementary file 1 [file Data_Sheet_1.PDF]

Supplementary Material

Peltigera frigida lichens and their substrates reduce the influence of forest cover change on phosphate solubilizing bacteria

Cecilia Muster[1], Diego Leiva[2], Camila Morales[1], Martin Grafe[3], Michael Schloter[3], Margarita Carú[1] and Julieta Orlando[1,4]

[1]Laboratory of Microbial Ecology, Facultad de Ciencias, Universidad de Chile, Chile

[2]Institute of Biology, University of Graz, Austria

[3]Research Unit Comparative Microbiome Analysis, Helmholtz Zentrum München, Germany

[4]Millennium Institute Biodiversity of Antarctic and Subantarctic Ecosystems (BASE), Chile

***Correspondence**:

Julieta Orlando. Laboratorio de Ecología Microbiana, Facultad de Ciencias, Universidad de Chile. Las Palmeras 3425, Ñuñoa, Santiago, Chile. Tel.: +56-2-29787401.
jorlando@uchile.cl

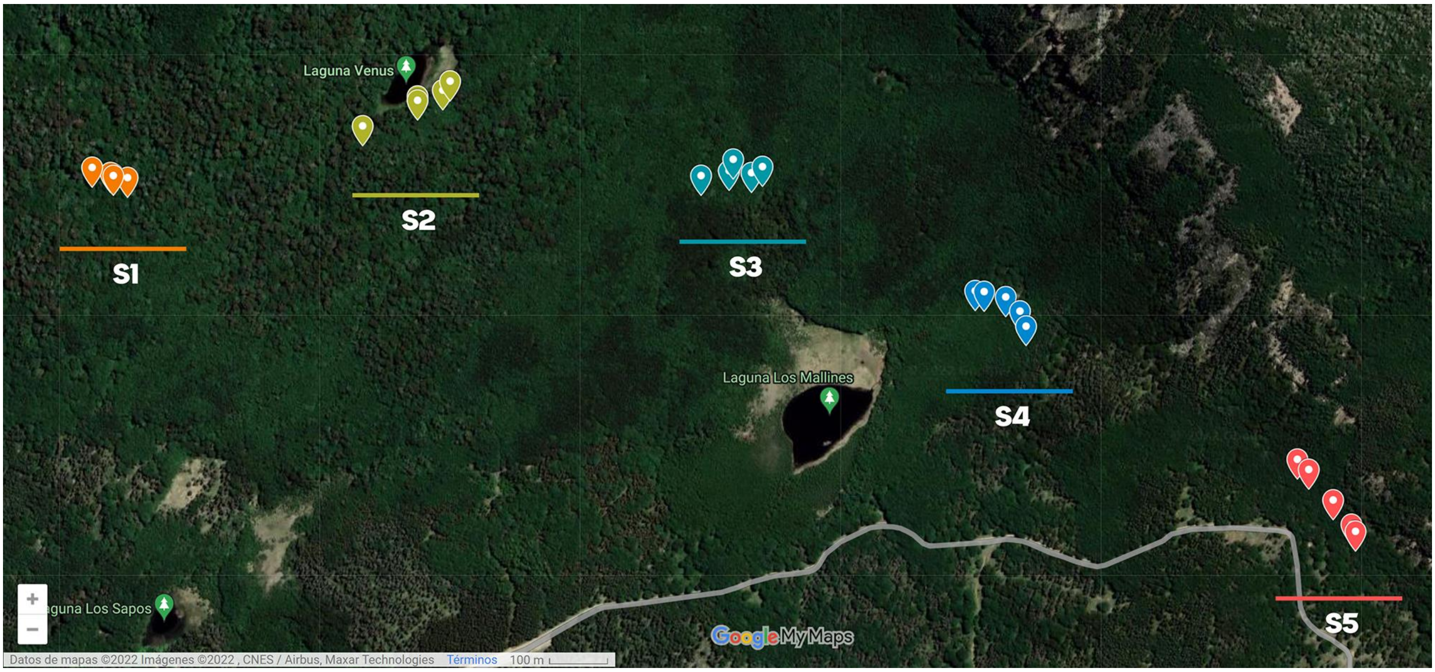

Figure S1. Map showing the geo-localization of *Peltigera frigida* samples in the five study sites in the Coyhaique National Reserve, Aysén Region, Chile.

Table S1. Inventory data and relative coverage (RC) values for the arboreal species *Nothofagus pumilio*. *Nothofagus dombeyi* and *Pinus contorta* obtained by the point intercept method for all sites.

Site	Species	p1	p2	p3	p4	p5	p6	p7	p8	p9	p10	p11	p12	p13	p14	p15	p16	p17	p18	p19	p20	RC
S1	*N. pumilio*	1	1	1	1	1		1	1	1		1	1	1	1	1	1	1	1	1	1	0.90
	N. dombeyi	1	1	1	1	1	1	1	1	1	1	1	1	1						1	1	0.75
	P. contorta																					0.00
S2	*N. pumilio*		1	1	1	1	1	1	1	1	1	1	1	1	1	1	1	-	-	-	-	0.94
	N. dombeyi	1			1	1		1	1	1	1	1				1	1	-	-	-	-	0.63
	P. contorta																	-	-	-	-	0.00
S3	*N. pumilio*	1	1		1	1		1	1	1	1	1	1	1	1	1	1	-	-	-	-	0.88
	N. dombeyi	1	1	1	1	1	1	1	1		1				1	1		-	-	-	-	0.69
	P. contorta																	-	-	-	-	0.00
S4	*N. pumilio*		1	1	1	1	1	1	1	1	1	1	1	1	1	1			-	-	-	0.82
	N. dombeyi	1	1	1	1				1	1				1	1	1	1	1	-	-	-	0.65
	P. contorta									1	1	1	1						-	-	-	0.24
S5	*N. pumilio*	1	1	1	1	1	1	1	1	1	1	1	1	1	1	1	1	1	-	-	-	1.00
	N. dombeyi				1	1	1												-	-	-	0.18
	P. contorta			1				1					1	1	1	1	1		-	-	-	0.41

Table S2. Field collected data and calculated values for the point-centered quarter method analysis for site S1.

	Field collected data				Calculated data		
Quadrant	Point	Closest species	Distance to reference point (cm)	Circumference at breast height (cm)	Radius (cm)	Area (cm^2)	Total density
I	p1	*N. pumilio*	340	45	7.2	161.2	0.06
	p2	*N. pumilio*	540	48	7.6	183.4	0.02
	p3	*N. pumilio*	390	88	14.0	616.6	0.04
	p4	*N. pumilio*	670	195	31.1	3027.5	0.01
	p5	*N. pumilio*	150	135	21.5	1451.0	0.28
	p6	*N. dombeyi*	250	41	6.5	133.8	0.10
	p7	*N. dombeyi*	140	100	15.9	796.2	0.33
	p8	*N. pumilio*	360	45	7.2	161.2	0.05
	p9	*N. pumilio*	270	28	4.5	62.4	0.09
	p10	*N. pumilio*	690	60	9.6	286.6	0.01
	p11	*N. pumilio*	520	50	8.0	199.0	0.02
	p12	*N. pumilio*	12	24	3.8	45.9	44.44
	p13	*N. pumilio*	1070	410	65.3	13383.8	0.01
	p14	*N. pumilio*	430	22	3.5	38.5	0.03
	p15	*N. pumilio*	700	332	52.9	8775.8	0.01
	p16	*N. pumilio*	764	160	25.5	2038.2	0.01
	p17	*N. pumilio*	150	39	6.2	121.1	0.28
	p18	*N. dombeyi*	550	26	4.1	53.8	0.02
	p19	*N. pumilio*	100	59	9.4	277.1	0.64
	p20	*N. pumilio*	470	34	5.4	92.0	0.03
II	p1	*N. pumilio*	260	44	7.0	154.1	0.09
	p2	*N. pumilio*	150	22	3.5	38.5	0.28
	p3	*N. pumilio*	490	57	9.1	258.7	0.03
	p4	*N. pumilio*	210	22	3.5	38.5	0.15
	p5	*N. pumilio*	230	25	4.0	49.8	0.12
	p6	*N. dombeyi*	142	84	13.4	561.8	0.32
	p7	*N. dombeyi*	520	68	10.8	368.2	0.02
	p8	*N. pumilio*	81	40	6.4	127.4	0.98
	p9	*N. pumilio*	400	118	18.8	1108.6	0.04
	p10	*N. dombeyi*	250	487	77.5	18882.9	0.10
	p11	*N. pumilio*	210	43	6.8	147.2	0.15
	p12	*N. pumilio*	30	17	2.7	23.0	7.11
	p13	*N. dombeyi*	210	360	57.3	10318.5	0.15
	p14	*N. pumilio*	510	9	1.4	6.4	0.02
	p15	*N. pumilio*	400	366	58.3	10665.3	0.04
	p16	*N. pumilio*	345	21	3.3	35.1	0.05
	p17	*N. pumilio*	320	30	4.8	71.7	0.06
	p18	*N. pumilio*	410	318	50.6	8051.3	0.04
	p19	*N. pumilio*	95	46	7.3	168.5	0.71
	p20	*N. pumilio*	260	28	4.5	62.4	0.09
III	p1	*N. dombeyi*	380	64	10.2	326.1	0.04
	p2	*N. pumilio*	140	23	3.7	42.1	0.33
	p3	*N. dombeyi*	200	172	27.4	2355.4	0.16
	p4	*N. pumilio*	510	52	8.3	215.3	0.02
	p5	*N. dombeyi*	200	12	1.9	11.5	0.16
	p6	*N. pumilio*	180	79	12.6	496.9	0.20
	p7	*N. dombeyi*	760	80	12.7	509.6	0.01
	p8	*N. pumilio*	130	58	9.2	267.8	0.38
	p9	*N. pumilio*	170	26	4.1	53.8	0.22
	p10	*N. pumilio*	400	28	4.5	62.4	0.04
	p11	*N. pumilio*	116	27	4.3	58.0	0.48
	p12	*N. pumilio*	60	23	3.7	42.1	1.78
	p13	*N. pumilio*	480	40	6.4	127.4	0.03
	p14	*N. dombeyi*	870	63	10.0	316.0	0.01
	p15	*N. pumilio*	570	174	27.7	2410.5	0.02
	p16	*N. pumilio*	129	43	6.8	147.2	0.38
	p17	*N. pumilio*	340	30	4.8	71.7	0.06
	p18	*N. pumilio*	300	35	5.6	97.5	0.07
	p19	*N. pumilio*	121	25	4.0	49.8	0.44
	p20	*N. pumilio*	80	35	5.6	97.5	1.00
IV	p1	*N. pumilio*	120	330	52.5	8670.4	0.44
	p2	*N. pumilio*	260	37	5.9	109.0	0.09
	p3	*N. pumilio*	240	52	8.3	215.3	0.11
	p4	*N. dombeyi*	300	143	22.8	1628.1	0.07
	p5	*N. pumilio*	430	20	3.2	31.8	0.03
	p6	*N. pumilio*	290	44	7.0	154.1	0.08
	p7	*N. dombeyi*	380	13	2.1	13.5	0.04
	p8	*N. dombeyi*	280	36	5.7	103.2	0.08
	p9	*N. pumilio*	70	35	5.6	97.5	1.31
	p10	*N. pumilio*	710	27	4.3	58.0	0.01
	p11	*N. pumilio*	270	38	6.1	115.0	0.09
	p12	*N. pumilio*	15	15	2.4	17.9	28.44
	p13	*N. dombeyi*	630	326	51.9	8461.5	0.02
	p14	*N. pumilio*	580	20	3.2	31.8	0.02
	p15	*N. dombeyi*	950	41	6.5	133.8	0.01
	p16	*N. pumilio*	270	26	4.1	53.8	0.09
	p17	*N. pumilio*	880	10	1.6	8.0	0.01
	p18	*N. pumilio*	400	35	5.6	97.5	0.04
	p19	*N. pumilio*	50	36	5.7	103.2	2.56
	p20	*N. pumilio*	410	41	6.5	133.8	0.04

Table S3. Field collected data and calculated values for the point-centered quarter method analysis for site S2.

Field collected data					Calculated data		
Quadrant	Point	Closest species	Distance to reference point (cm)	Circumference at breast height (cm)	Radius (cm)	Area (cm^2)	Total density
I	p1	*N. pumilio*	160	17	2.7	23.0	0.25
	p2	*N. pumilio*	460	56	8.9	249.7	0.03
	p3	*N. dombeyi*	270	15	2.4	17.9	0.09
	p4	*N. pumilio*	650	110	17.5	963.4	0.02
	p5	*N. pumilio*	470	15	2.4	17.9	0.03
	p6	*N. pumilio*	60	45	7.2	161.2	1.78
	p7	*N. dombeyi*	320	220	35.0	3853.5	0.06
	p8	*N. pumilio*	320	31	4.9	76.5	0.06
	p9	*N. dombeyi*	440	48	7.6	183.4	0.03
	p10	*N. dombeyi*	105	12	1.9	11.5	0.58
	p11	*N. pumilio*	420	26	4.1	53.8	0.04
	p12	*N. pumilio*	46	12	1.9	11.5	3.02
	p13	*N. pumilio*	520	36	5.7	103.2	0.02
	p14	*N. pumilio*	34	52	8.3	215.3	5.54
	p15	*N. pumilio*	650	79	12.6	496.9	0.02
	p16	*N. pumilio*	520	42	6.7	140.4	0.02
II	p1	*N. dombeyi*	240	127	20.2	1284.2	0.11
	p2	*N. pumilio*	100	68	10.8	368.2	0.64
	p3	*N. pumilio*	500	206	32.8	3378.7	0.03
	p4	*N. dombeyi*	360	480	76.4	18343.9	0.05
	p5	*N. dombeyi*	480	240	38.2	4586.0	0.03
	p6	*N. pumilio*	42	48	7.6	183.4	3.63
	p7	*N. pumilio*	210	38	6.1	115.0	0.15
	p8	*N. pumilio*	150	55	8.8	240.8	0.28
	p9	*N. pumilio*	90	7	1.1	3.9	0.79
	p10	*N. dombeyi*	200	2	0.3	0.3	0.16
	p11	*N. pumilio*	200	33	5.3	86.7	0.16
	p12	*N. pumilio*	40	6	1.0	2.9	4.00
	p13	*N. pumilio*	210	34	5.4	92.0	0.15
	p14	*N. pumilio*	88	9	1.4	6.4	0.83
	p15	*N. pumilio*	530	47	7.5	175.9	0.02
	p16	*N. dombeyi*	170	25	4.0	49.8	0.22
III	p1	*N. pumilio*	260	14	2.2	15.6	0.09
	p2	*N. pumilio*	150	52	8.3	215.3	0.28
	p3	*N. pumilio*	190	286	45.5	6512.4	0.18
	p4	*N. pumilio*	400	32	5.1	81.5	0.04
	p5	*N. pumilio*	190	255	40.6	5177.1	0.18
	p6	*N. pumilio*	80	36	5.7	103.2	1.00
	p7	*N. pumilio*	130	32	5.1	81.5	0.38
	p8	*N. pumilio*	240	50	8.0	199.0	0.11
	p9	*N. pumilio*	260	13	2.1	13.5	0.09
	p10	*N. pumilio*	170	9	1.4	6.4	0.22
	p11	*N. pumilio*	40	11	1.8	9.6	4.00
	p12	*N. pumilio*	50	4	0.6	1.3	2.56
	p13	*N. pumilio*	230	197	31.4	3089.9	0.12
	p14	*N. pumilio*	77	9	1.4	6.4	1.08
	p15	*N. dombeyi*	270	324	51.6	8358.0	0.09
	p16	*N. pumilio*	50	40	6.4	127.4	2.56
IV	p1	*N. pumilio*	160	11	1.8	9.6	0.25
	p2	*N. pumilio*	840	22	3.5	38.5	0.01
	p3	*N. pumilio*	610	55	8.8	240.8	0.02
	p4	*N. pumilio*	640	63	10.0	316.0	0.02
	p5	*N. pumilio*	510	51	8.1	207.1	0.02
	p6	*N. pumilio*	60	50	8.0	199.0	1.78
	p7	*N. pumilio*	420	15	2.4	17.9	0.04
	p8	*N. dombeyi*	108	33	5.3	86.7	0.55
	p9	*N. dombeyi*	530	265	42.2	5591.2	0.02
	p10	*N. dombeyi*	100	5	0.8	2.0	0.64
	p11	*N. pumilio*	410	58	9.2	267.8	0.04
	p12	*N. pumilio*	35	11	1.8	9.6	5.22
	p13	*N. pumilio*	520	30	4.8	71.7	0.02
	p14	*N. pumilio*	44	34	5.4	92.0	3.31
	p15	*N. pumilio*	570	35	5.6	97.5	0.02
	p16	*N. pumilio*	95	51	8.1	207.1	0.71

Table S4. Field collected data and calculated values for the point-centered quarter method analysis for site S3.

Field collected data					Calculated data		
Quadrant	Point	Closest species	Distance to reference point (cm)	Circumference at breast height (cm)	Radius (cm)	Area (cm^2)	Total density
I	p1	*N. pumilio*	107	34	5.4	92.0	0.56
	p2	*N. dombeyi*	600	41	6.5	133.8	0.02
	p3	*N. pumilio*	390	47	7.5	175.9	0.04
	p4	*N. pumilio*	420	64	10.2	326.1	0.04
	p5	*N. pumilio*	69	30	4.8	71.7	1.34
	p6	*N. pumilio*	470	25	4.0	49.8	0.03
	p7	*N. pumilio*	66	56	8.9	249.7	1.47
	p8	*N. dombeyi*	160	125	19.9	1244.0	0.25
	p9	*N. pumilio*	180	31	4.9	76.5	0.20
	p10	*N. pumilio*	240	23	3.7	42.1	0.11
	p11	*N. pumilio*	89	47	7.5	175.9	0.81
	p12	*N. pumilio*	82	50	8.0	199.0	0.95
	p13	*N. pumilio*	77	32	5.1	81.5	1.08
	p14	*N. pumilio*	61	19	3.0	28.7	1.72
	p15	*N. dombeyi*	220	51	8.1	207.1	0.13
	p16	*N. pumilio*	84	67	10.7	357.4	0.91
II	p1	*N. pumilio*	82	34	5.4	92.0	0.95
	p2	*N. pumilio*	130	33	5.3	86.7	0.38
	p3	*N. dombeyi*	140	270	43.0	5804.1	0.33
	p4	*N. pumilio*	81	82	13.1	535.4	0.98
	p5	*N. dombeyi*	270	40	6.4	127.4	0.09
	p6	*N. pumilio*	260	48	7.6	183.4	0.09
	p7	*N. pumilio*	330	129	20.5	1324.9	0.06
	p8	*N. pumilio*	260	62	9.9	306.1	0.09
	p9	*N. pumilio*	190	34	5.4	92.0	0.18
	p10	*N. pumilio*	130	76	12.1	459.9	0.38
	p11	*N. pumilio*	114	30	4.8	71.7	0.49
	p12	*N. pumilio*	55	68	10.8	368.2	2.12
	p13	*N. pumilio*	60	54	8.6	232.2	1.78
	p14	*N. pumilio*	63	22	3.5	38.5	1.61
	p15	*N. pumilio*	150	64	10.2	326.1	0.28
	p16	*N. pumilio*	230	135	21.5	1451.0	0.12
III	p1	*N. pumilio*	78	33	5.3	86.7	1.05
	p2	*N. dombeyi*	190	120	19.1	1146.5	0.18
	p3	*N. dombeyi*	610	175	27.9	2438.3	0.02
	p4	*N. pumilio*	220	60	9.6	286.6	0.13
	p5	*N. pumilio*	200	37	5.9	109.0	0.16
	p6	*N. dombeyi*	300	68	10.8	368.2	0.07
	p7	*N. pumilio*	370	101	16.1	812.2	0.05
	p8	*N. pumilio*	580	108	17.2	928.7	0.02
	p9	*N. pumilio*	260	68	10.8	368.2	0.09
	p10	*N. pumilio*	240	76	12.1	459.9	0.11
	p11	*N. pumilio*	66	28	4.5	62.4	1.47
	p12	*N. pumilio*	88	41	6.5	133.8	0.83
	p13	*N. pumilio*	170	82	13.1	535.4	0.22
	p14	*N. pumilio*	240	38	6.1	115.0	0.11
	p15	*N. pumilio*	260	26	4.1	53.8	0.09
	p16	*N. pumilio*	98	46	7.3	168.5	0.67
IV	p1	*N. pumilio*	240	38	6.1	115.0	0.11
	p2	*N. pumilio*	750	76	12.1	459.9	0.01
	p3	*N. pumilio*	530	69	11.0	379.1	0.02
	p4	*N. pumilio*	260	86	13.7	588.9	0.09
	p5	*N. dombeyi*	220	47	7.5	175.9	0.13
	p6	*N. dombeyi*	90	166	26.4	2193.9	0.79
	p7	*N. dombeyi*	74	79	12.6	496.9	1.17
	p8	*N. pumilio*	200	88	14.0	616.6	0.16
	p9	*N. pumilio*	61	65	10.4	336.4	1.72
	p10	*N. pumilio*	150	53	8.4	223.6	0.28
	p11	*N. pumilio*	105	31	4.9	76.5	0.58
	p12	*N. pumilio*	135	46	7.3	168.5	0.35
	p13	*N. pumilio*	250	36	5.7	103.2	0.10
	p14	*N. pumilio*	290	26	4.1	53.8	0.08
	p15	*N. pumilio*	81	62	9.9	306.1	0.98
	p16	*N. pumilio*	143	36	5.7	103.2	0.31

Table S5. Field collected data and calculated values for the point-centered quarter method analysis for site S4.

	Field collected data				Calculated data		
Quadrant	Point	Closest species	Distance to reference point (cm)	Circumference at breast height (cm)	Radius (cm)	Area (cm^2)	Total density
I	p1	*N. dombeyi*	380	61	9.7	296.3	0.04
	p2	*N. pumilio*	320	128	20.4	1304.5	0.06
	p3	*N. pumilio*	98	112	17.8	998.7	0.67
	p4	*N. pumilio*	280	143	22.8	1628.1	0.08
	p5	*N. pumilio*	91	20	3.2	31.8	0.77
	p6	*N. pumilio*	150	110	17.5	963.4	0.28
	p7	*N. pumilio*	70	104	16.6	861.1	1.31
	p8	*N. pumilio*	48	40	6.4	127.4	2.78
	p9	*N. pumilio*	230	50	8.0	199.0	0.12
	p10	*N. pumilio*	320	84	13.4	561.8	0.06
	p11	*N. pumilio*	55	14	2.2	15.6	2.12
	p12	*P. contorta*	270	50	8.0	199.0	0.09
	p13	*P. contorta*	540	114	18.2	1034.7	0.02
	p14	*N. dombeyi*	190	52	8.3	215.3	0.18
	p15	*N. dombeyi*	730	391	62.3	12172.1	0.01
	p16	*N. pumilio*	1060	395	62.9	12422.4	0.01
	p17	*P. contorta*	330	47	7.5	175.9	0.06
II	p1	*N. dombeyi*	390	98	15.6	764.6	0.04
	p2	*N. pumilio*	300	106	16.9	894.6	0.07
	p3	*N. dombeyi*	280	95	15.1	718.6	0.08
	p4	*N. pumilio*	180	55	8.8	240.8	0.20
	p5	*N. pumilio*	142	42	6.7	140.4	0.32
	p6	*N. pumilio*	80	150	23.9	1791.4	1.00
	p7	*N. pumilio*	100	32	5.1	81.5	0.64
	p8	*N. dombeyi*	210	41	6.5	133.8	0.15
	p9	*P. contorta*	96	75	11.9	447.9	0.69
	p10	*N. pumilio*	260	182	29.0	2637.3	0.09
	p11	*N. pumilio*	53	67	10.7	357.4	2.28
	p12	*N. dombeyi*	360	25	4.0	49.8	0.05
	p13	*N. pumilio*	240	170	27.1	2301.0	0.11
	p14	*N. pumilio*	300	232	36.9	4285.4	0.07
	p15	*N. dombeyi*	590	63	10.0	316.0	0.02
	p16	*N. pumilio*	351	265	42.2	5591.2	0.05
	p17	*N. dombeyi*	190	142	22.6	1605.4	0.18
III	p1	*N. pumilio*	240	27	4.3	58.0	0.11
	p2	*N. dombeyi*	370	81	12.9	522.4	0.05
	p3	*N. pumilio*	150	46	7.3	168.5	0.28
	p4	*N. dombeyi*	65	42	6.7	140.4	1.51
	p5	*N. pumilio*	80	16	2.5	20.4	1.00
	p6	*N. dombeyi*	90	23	3.7	42.1	0.79
	p7	*N. pumilio*	150	23	3.7	42.1	0.28
	p8	*N. pumilio*	35	67	10.7	357.4	5.22
	p9	*P. contorta*	190	65	10.4	336.4	0.18
	p10	*N. pumilio*	200	30	4.8	71.7	0.16
	p11	*P. contorta*	180	49	7.8	191.2	0.20
	p12	*P. contorta*	280	67	10.7	357.4	0.08
	p13	*N. dombeyi*	155	76	12.1	459.9	0.27
	p14	*N. dombeyi*	220	20	3.2	31.8	0.13
	p15	*N. pumilio*	690	174	27.7	2410.5	0.01
	p16	*N. dombeyi*	150	32	5.1	81.5	0.28
	p17	*N. dombeyi*	380	355	56.5	10033.8	0.04
IV	p1	*N. dombeyi*	52	92	14.6	673.9	2.37
	p2	*N. dombeyi*	330	64	10.2	326.1	0.06
	p3	*N. dombeyi*	86	59	9.4	277.1	0.87
	p4	*N. pumilio*	170	40	6.4	127.4	0.22
	p5	*N. pumilio*	70	15	2.4	17.9	1.31
	p6	*N. dombeyi*	190	90	14.3	644.9	0.18
	p7	*N. pumilio*	130	65	10.4	336.4	0.38
	p8	*N. pumilio*	66	111	17.7	981.0	1.47
	p9	*N. dombeyi*	250	85	13.5	575.2	0.10
	p10	*P. contorta*	120	98	15.6	764.6	0.44
	p11	*N. pumilio*	240	79	12.6	496.9	0.11
	p12	*P. contorta*	300	42	6.7	140.4	0.07
	p13	*N. pumilio*	116	100	15.9	796.2	0.48
	p14	*P. contorta*	320	47	7.5	175.9	0.06
	p15	*N. dombeyi*	390	277	44.1	6109.0	0.04
	p16	*N. pumilio*	826	86	13.7	588.9	0.01
	p17	*P. contorta*	670	68	10.8	368.2	0.01

Table S6. Field collected data and calculated values for the point-centered quarter method analysis for site S5.

Field collected data					Calculated data		
Quadrant	Point	Closest species	Distance to reference point (cm)	Circumference at chest height (cm)	Radius (cm)	Area (cm^2)	Total density
I	p1	*N. pumilio*	210	87	13.9	602.6	0.15
	p2	*N. pumilio*	560	402	64.0	12866.6	0.02
	p3	*P. contorta*	270	97	15.4	749.1	0.09
	p4	*N. pumilio*	230	227	36.1	4102.6	0.12
	p5	*N. pumilio*	76	159	25.3	2012.8	1.11
	p6	*N. pumilio*	220	115	18.3	1052.9	0.13
	p7	*P. contorta*	130	62	9.9	306.1	0.38
	p8	*N. pumilio*	400	26	4.1	53.8	0.04
	p9	*N. pumilio*	112	218	34.7	3783.8	0.51
	p10	*P. contorta*	360	44	7.0	154.1	0.05
	p11	*N. pumilio*	360	2	0.3	0.3	0.05
	p12	*P. contorta*	85	75	11.9	447.9	0.89
	p13	*P. contorta*	380	87	13.9	602.6	0.04
	p14	*P. contorta*	96	75	11.9	447.9	0.69
	p15	*P. contorta*	570	64	10.2	326.1	0.02
	p16	*P. contorta*	340	87	13.9	602.6	0.06
	p17	*N. dombeyi*	397	119	18.9	1127.5	0.04
II	p1	*N. pumilio*	70	64	10.2	326.1	1.31
	p2	*N. pumilio*	340	86	13.7	588.9	0.06
	p3	*N. pumilio*	260	107	17.0	911.5	0.09
	p4	*N. dombeyi*	180	58	9.2	267.8	0.20
	p5	*N. dombeyi*	220	93	14.8	688.6	0.13
	p6	*N. pumilio*	120	79	12.6	496.9	0.44
	p7	*N. pumilio*	190	23	3.7	42.1	0.18
	p8	*N. pumilio*	100	129	20.5	1324.9	0.64
	p9	*N. pumilio*	200	86	13.7	588.9	0.16
	p10	*N. pumilio*	120	67	10.7	357.4	0.44
	p11	*N. dombeyi*	380	113	18.0	1016.6	0.04
	p12	*N. pumilio*	590	152	24.2	1839.5	0.02
	p13	*P. contorta*	230	50	8.0	199.0	0.12
	p14	*P. contorta*	133	95	15.1	718.6	0.36
	p15	*P. contorta*	88	114	18.2	1034.7	0.83
	p16	*N. dombeyi*	307	131	20.9	1366.3	0.07
	p17	*N. pumilio*	100	226	36.0	4066.6	0.64
III	p1	*N. pumilio*	210	169	26.9	2274.0	0.15
	p2	*N. pumilio*	820	294	46.8	6881.8	0.01
	p3	*P. contorta*	280	97	15.4	749.1	0.08
	p4	*N. dombeyi*	170	22	3.5	38.5	0.22
	p5	*N. pumilio*	100	197	31.4	3089.9	0.64
	p6	*N. pumilio*	150	142	22.6	1605.4	0.28
	p7	*N. pumilio*	137	158	25.2	1987.6	0.34
	p8	*N. pumilio*	240	65	10.4	336.4	0.11
	p9	*N. pumilio*	250	77	12.3	472.1	0.10
	p10	*N. pumilio*	260	174	27.7	2410.5	0.09
	p11	*N. pumilio*	590	225	35.8	4030.7	0.02
	p12	*N. pumilio*	250	150	23.9	1791.4	0.10
	p13	*P. contorta*	175	125	19.9	1244.0	0.21
	p14	*N. pumilio*	170	60	9.6	286.6	0.22
	p15	*P. contorta*	260	76	12.1	459.9	0.09
	p16	*N. pumilio*	327	324	51.6	8358.0	0.06
	p17	*N. pumilio*	250	107	17.0	911.5	0.10
IV	p1	*N. pumilio*	460	55	8.8	240.8	0.03
	p2	*N. pumilio*	280	378	60.2	11376.1	0.08
	p3	*N. pumilio*	410	163	26.0	2115.4	0.04
	p4	*N. dombeyi*	370	71	11.3	401.4	0.05
	p5	*N. pumilio*	94	28	4.5	62.4	0.72
	p6	*N. pumilio*	112	36	5.7	103.2	0.51
	p7	*P. contorta*	90	79	12.6	496.9	0.79
	p8	*N. pumilio*	300	46	7.3	168.5	0.07
	p9	*N. pumilio*	140	73	11.6	424.3	0.33
	p10	*N. pumilio*	40	62	9.9	306.1	4.00
	p11	*N. pumilio*	60	100	15.9	796.2	1.78
	p12	*N. pumilio*	380	241	38.4	4624.3	0.04
	p13	*P. contorta*	410	48	7.6	183.4	0.04
	p14	*N. pumilio*	410	168	26.8	2247.1	0.04
	p15	*N. pumilio*	75	128	20.4	1304.5	1.14
	p16	*P. contorta*	86	89	14.2	630.7	0.87
	p17	*N. pumilio*	460	141	22.5	1582.9	0.03

Table S7. Results for the point-centered quarter method of tree species *Nothofagus pumilio*. *Nothofagus dombeyi* and *Pinus contorta* for all sites. Calculated values shown here include frequency, relative frequency, coverage, relative coverage, and importance value.

Indice	Site	*N. pumilio*	*N. dombeyi*	*P. contorta*
Frequency	S1	0.95	0.60	0.00
	S2	1.00	0.63	0.00
	S3	1.00	0.44	0.00
	S4	0.88	0.77	0.41
	S5	0.94	0.29	0.47
Relative frequency	S1	0.61	0.39	0.00
	S2	0.62	0.39	0.00
	S3	0.70	0.30	0.00
	S4	0.43	0.37	0.20
	S5	0.55	0.17	0.28
Coverage	S1	78307.3	923.5	0.0
	S2	17608.1	1743.0	0.0
	S3	6762.4	710.3	0.0
	S4	15587.0	3959.2	117.8
	S5	23910.8	54.2	770.7
Relative coverage	S1	0.99	0.01	0.00
	S2	0.91	0.09	0.00
	S3	0.90	0.10	0.00
	S4	0.79	0.20	0.01
	S5	0.97	0.00	0.03
Importance value	S1	2.39	0.61	0.00
	S2	2.32	0.68	0.00
	S3	2.43	0.57	0.00
	S4	1.74	0.90	0.37
	S5	2.17	0.28	0.56

Table S8. Nomenclature and geo-localization information of *Peltigera frigida* samples in the Coyhaique National Reserve, Aysén Region, Chile.

Site	Lichens	Collection date	Latitude (°)	Longitude (°)	Altitude (m)	GBIF occurrence[1]
S1	L01	2018	-45.528	-72.047	700	3064115729
	L02	2018	-45.528	-72.047	701	3064115731
	L03	2018	-45.528	-72.047	701	3064115727
	L04	2018	-45.528	-72.048	702	3064115726
	L05	2016	-45.528	-72.047	697	3064116265
S2	L06	2018	-45.527	-72.041	685	3064115728
	L07	2016	-45.527	-72.042	740	3064116143
	L08	2016	-45.527	-72.041	735	3064116182
	L09	2016	-45.526	-72.040	733	3064116110
	L10	2016	-45.526	-72.040	733	3064116130
S3	L11	2018	-45.528	-72.034	656	3064115725
	L12	2018	-45.528	-72.034	657	3064115720
	L13	2018	-45.528	-72.034	656	3064115724
	L14	2013	-45.527	-72.034	708	3064115372
	L15	2013	-45.528	-72.035	713	3064115357
S4	L16	2018	-45.529	-72.029	649	3064115631
	L17	2018	-45.529	-72.029	649	3064115632
	L18	2018	-45.529	-72.028	651	3064115630
	L19	2018	-45.530	-72.028	655	3064115633
	L20	2018	-45.530	-72.028	656	3064115635
S5	L21	2018	-45.532	-72.021	654	3064115645
	L22	2018	-45.533	-72.021	647	3064115643
	L23	2016	-45.532	-72.022	679	3064116138
	L24	2016	-45.533	-72.022	724	3064116115
	L25	2016	-45.533	-72.021	724	3064116298

[1] In: Orlando J, Almendras K, Veas-Mattheos-Mattheos K, Pezoa M, Carú M. 2021. *Peltigera* cyanolichens from Southern Chile. Version 1.4. Laboratory of Microbial Ecology (Laboratorio de Ecología Microbiana) - Universidad de Chile. Occurrence dataset https://doi.org/10.15468/ef76hj accessed via GBIF.org.

Table S9. Nomenclature and geo-localization information of soil samples in the Coyhaique National Reserve, Aysén Region, Chile.

Site	Subsamples	Latitude (°)	Longitude (°)	Altitude (m)
S1	S1.1	-45,5276	-72,0499	700
	S1.2	-45,5279	-72,0492	701
	S1.3	-45,5280	-72,0489	701
	S1.4	-45,5275	-72,0477	702
	S1.5	-45,5276	-72,0469	697
S2	S2.1	-45,5272	-72,0427	685
	S2.2	-45,5268	-72,0421	740
	S2.3	-45,5266	-72,0412	735
	S2.4	-45,5263	-72,0401	733
	S2.5	-45,5263	-72,0397	733
S3	S3.1	-45,5275	-72,0363	642
	S3.2	-45,5277	-72,0356	635
	S3.3	-45,5277	-72,0349	632
	S3.4	-45,5276	-72,0340	631
	S3.5	-45,5276	-72,0332	630
S4	S4.1	-45,5293	-72,0299	617
	S4.2	-45,5293	-72,0293	624
	S4.3	-45,5294	-72,0286	630
	S4.4	-45,5297	-72,0280	636
	S4.5	-45,5296	-72,0274	640
S5	S5.1	-45,5320	-72,0220	639
	S5.2	-45,5321	-72,0220	639
	S5.3	-45,5324	-72,0216	637
	S5.4	-45,5328	-72,0211	630
	S5.5	-45,5332	-72,0207	626

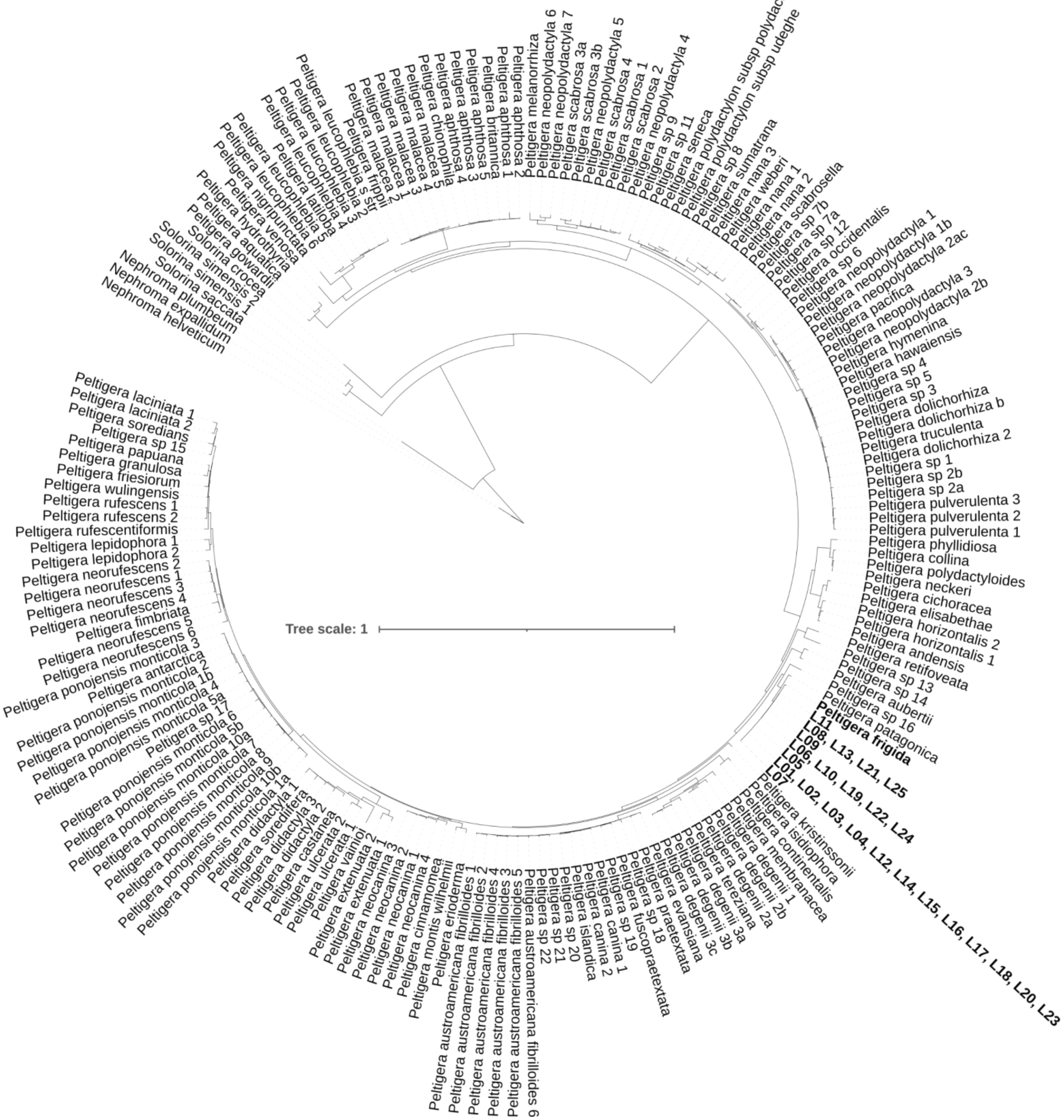

Fig. S2. Phylogenetic tree of mycobiont sequences of genus *Peltigera* analyzed in the T-BAS platform based on 28S rRNA gene and ITS region sequences and edited in iTOL. The samples of this work (L01-L25) are highlighted with bold.

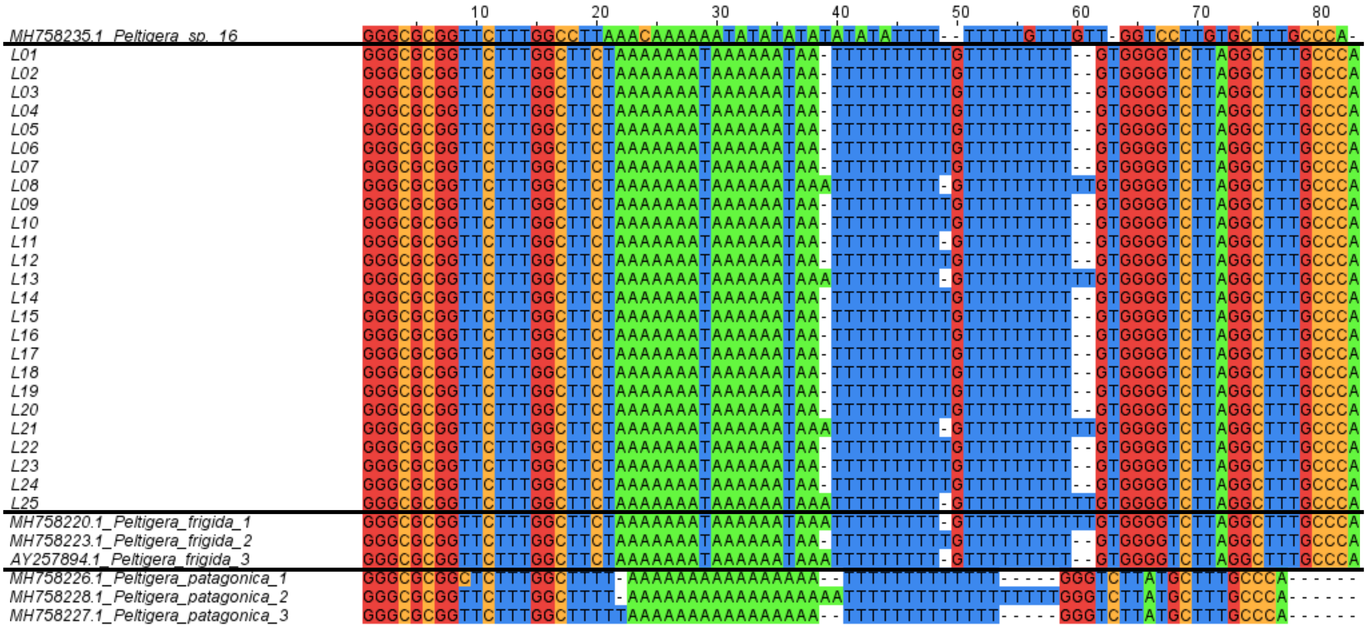

Fig. S3. Alignment of the ITS hypervariable region (ITS-HR) of the 25 *Peltigera frigida* lichen samples from this work (L01 to L25) and those published by Magain et al. [2] for *Peltigera* sp. 16. *P. frigida* and *P. patagonica* species.

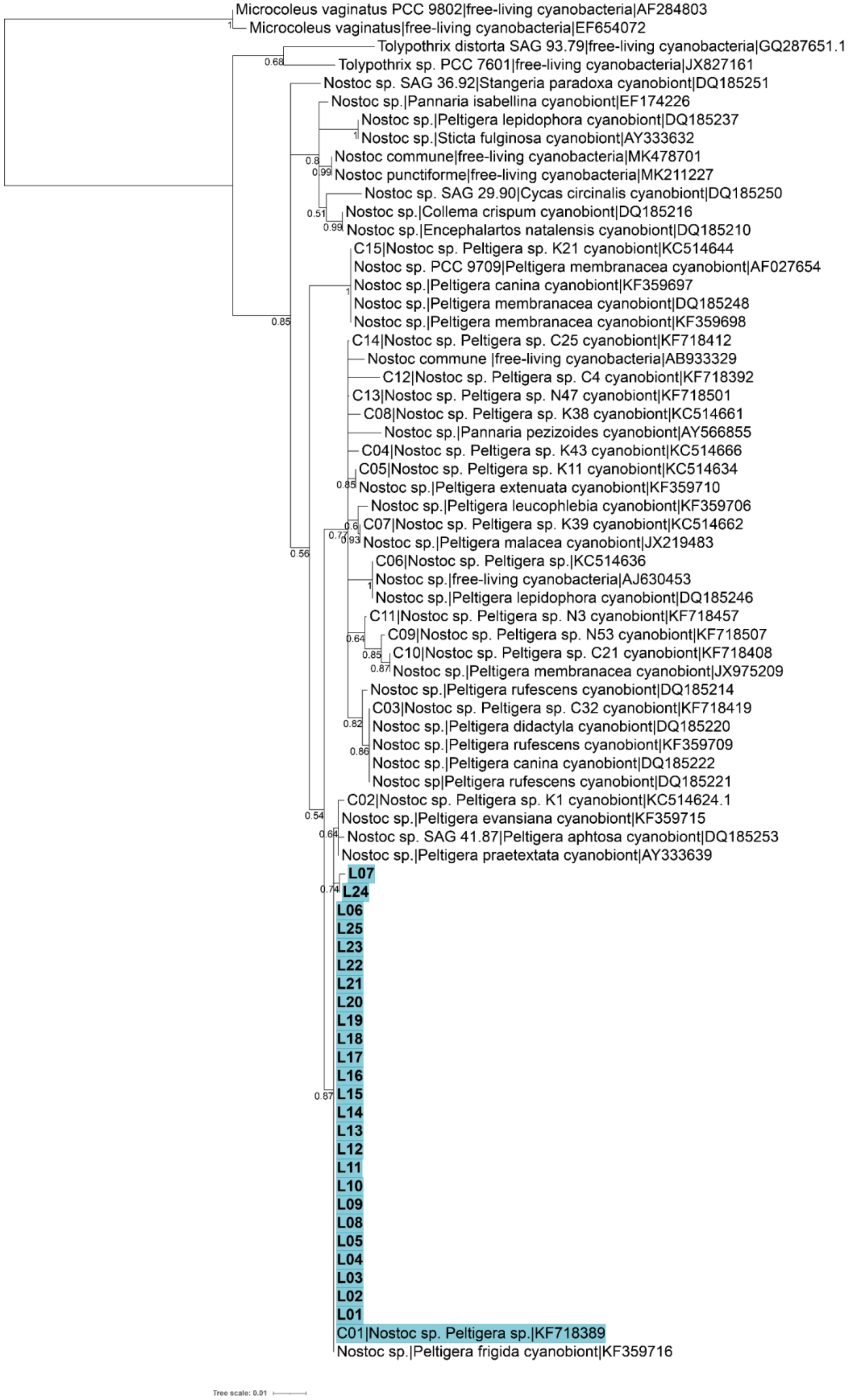

Fig. S4. Phylogenetic tree of cyanobiont 16S rRNA gene sequences from lichen samples using Neighbor-Joining. The analysis included 15 haplotypes found by Zúñiga et al. (2015) associated with lichens of the genus *Peltigera*. 30 reference sequences of *Nostoc* obtained from cyanolichens. plants. and free-living cyanobacteria and 25 sequences of this work (L01-L25 in bold and blue background).Four sequences belonging to the genera *Tolypothrix* (2) and *Microcoleus* (2) were used as external groups.

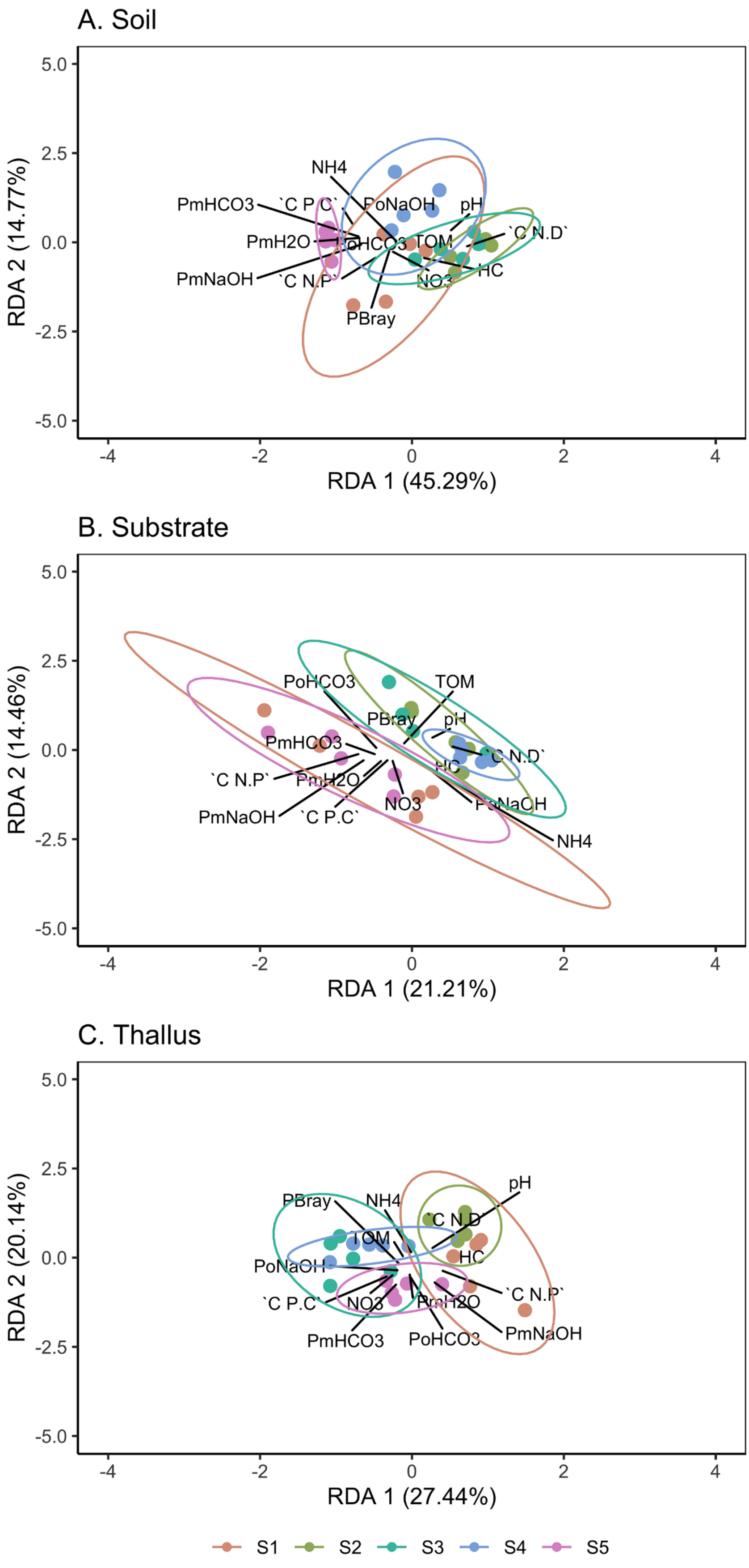

Fig. S5. Redundancy Analysis (RDA) of the abundance of phosphate solubilization and mineralization markers for each site. (A) Soil samples. (B) *Peltigera frigida* substrate samples. (C) *Peltigera frigida* thallus samples. 95% ellipses are included for each site.
